# Relationships between pathology and crystal structure in breast calcifications: an *in situ* X-ray diffraction study in histological sections

**DOI:** 10.1038/npjbcancer.2016.29

**Published:** 2016-09-14

**Authors:** Robert Scott, Nicholas Stone, Catherine Kendall, Kalotina Geraki, Keith Rogers

**Affiliations:** 1 Biophotonics Research Unit, Gloucestershire Royal Hospital, Gloucester, UK; 2 School of Physics, University of Exeter, Exeter, UK; 3 Diamond Light Source, Didcot, UK; 4 Cranfield Forensic Institute, Cranfield University, Cranfield, UK

## Abstract

Calcifications are not only one of the most important early diagnostic markers of breast cancer, but are also increasingly believed to aggravate the proliferation of cancer cells and invasion of surrounding tissue. Moreover, this influence appears to vary with calcification composition. Despite this, remarkably little is known about the composition and crystal structure of the most common type of breast calcifications, and how this differs between benign and malignant lesions. We sought to determine how the phase composition and crystallographic parameters within calcifications varies with pathology, using synchrotron X-ray diffraction. This is the first time crystallite size and lattice parameters have been measured in breast calcifications, and we found that these both parallel closely the changes in these parameters with age observed in fetal bone. We also discovered that these calcifications contain a small proportion of magnesium whitlockite, and that this proportion increases from benign to *in situ* to invasive cancer. When combined with other recent evidence on the effect of magnesium on hydroxyapatite precipitation, this suggests a mechanism explaining observations that carbonate levels within breast calcifications are lower in malignant specimens.

## Introduction

Despite the central role of calcifications in the early detection of breast cancer, their pathophysiology and chemistry remain poorly understood.^1–3^ This is important for two reasons:

First, there is evidence that calcifications in breast cancer are not simply passive products of the disease process, but may have an active role in mitogenesis, upregulation of gene expression and enhanced migration of tumor cells.^1,4,5^ In particular, calcifications associated with breast cancer consist predominantly of hydroxyapatite, and there is evidence that nanoscale properties of hydroxyapatite may have an important role in regulating breast cancer cell behavior.^6^ Understanding the nature of calcifications, and how and why they form, is important in developing a complete picture of the factors affecting tumor development and progression.

Second, calcification chemistry has potential for use in diagnosis, either as an objective adjunct to conventional histopathology, or by noninvasive probing, e.g., using Raman spectroscopy.^7–9^ Given that mammography screening only has a 20% positive predictive value,^10^ and that in breast pathology ‘Many gradations of normal, precancerous, and cancerous conditions are often indistinguishable, making clear-cut diagnosis very difficult and often a cause for intense debate’,^11^ accurate, rapid, and objective aids to diagnosis clearly have a role.^12^

For both these purposes, it is necessary to determine and explain the relationship between calcification chemistry and pathology. Early studies on calcifications dissected from fresh excised breast tissue revealed the presence of two distinct chemical and crystallographic types: ‘Type I’^13^ are composed of calcium oxalate dihydrate in the crystalline form of weddellite, and are comparatively rare. The prevailing consensus is that deposition is a feature of benign rather than malignant processes within the breast.^4,14^ ‘Type II’ are much more common, and are composed of calcium phosphates with an apatite structure. These can occur in both benign and malignant lesions. More recent quantitative investigations into the chemistry of Type II calcifications and its relationship with pathology have largely used Raman spectroscopy or FTIR (Fourier transform infrared) spectroscopy. An important finding is that the proportion of carbonate substitution in the hydroxyapatite lattice is higher in benign than malignant specimens.^15^ Moreover, the carbonate level decreases sequentially from benign to ductal carcinoma *in situ*, to invasive cancer, and in the carcinoma specimens, carbonate substitution is negatively correlated with histological grade.^16^ This finding has potential uses in Raman discrimination of pathology.

However, there are limitations to these previous studies that need to be addressed to progress further towards analysis of calcification chemistry as a diagnostic tool. Although vibrational spectroscopy gives useful information on the nature, concentration, and environment of the anions (e.g., phosphate, carbonate, hydroxide), it gives almost no information on the cations present (principally calcium). Any chemical characterization of calcifications without reference to calcium cannot be regarded as complete.

In this study, we have sought to address these gaps by quantitative X-ray diffraction analysis of individual calcifications. This technique gives information on the structure of the crystalline phases comprising the calcifications, which can then be related to the pathology of the specimens. To date, X-ray diffraction of breast calcifications has been limited to a qualitative identification of major phases present in small numbers of samples. There have been no quantitative investigations of crystal lattice parameters and peak broadening, which provide information concerning ionic substitutions and crystallographic disorder, respectively. Moreover, investigations to date have relied on dissection of calcifications from bulk tissue specimens. Any analysis relying on fresh tissue is hampered by the difficulty of accessing a wide range of specimens. In contrast, diagnostic archives represent a rich source of material with varying pathology, and histological sections cut from archive blocks are readily available.

It is technically challenging to acquire spatially resolved X-ray diffraction data of sufficient quality for quantitative analysis from the very small volumes of poorly crystalline material present in these histological sections. To the best of our knowledge, *in situ* X-ray diffraction analysis on histological sections of any sort has only been reported twice before,^17,18^ in studies of vascular calcifications; this is the first time such a technique has been used to study breast calcifications. The techniques developed could readily be used for investigation of other pathological calcifications; similarities and differences may shed light on the mechanisms by which these are formed.

## Results

### Phase composition

**Figure 1:**
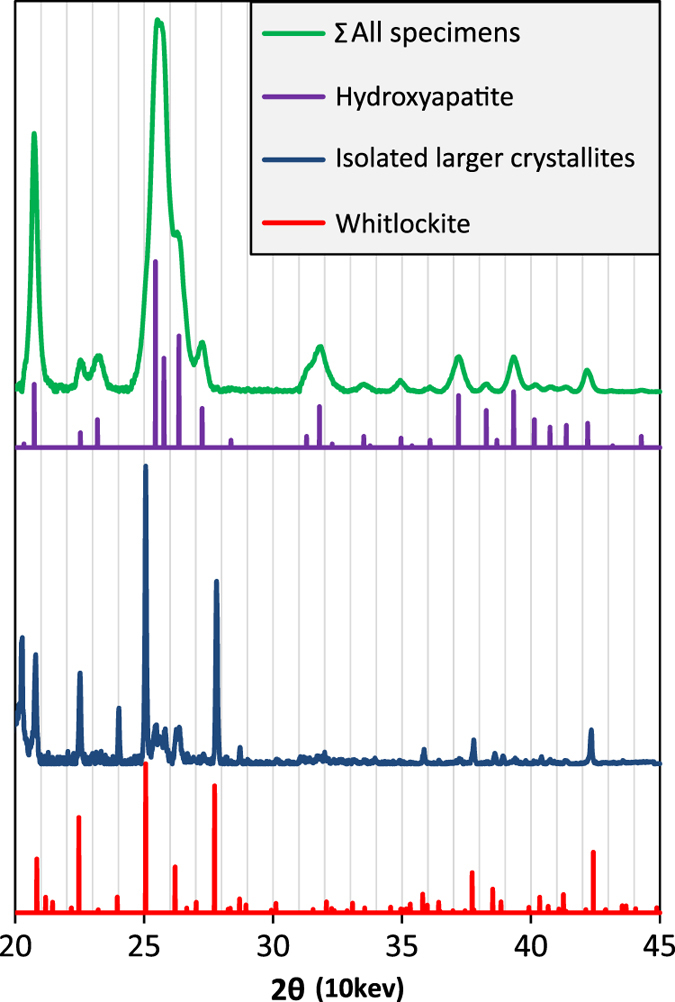
All-specimens sum diffractogram and digitally isolated large crystallite diffractogram versus theoretical line positions and intensities.

The predominant phase in these calcifications is nanocrystalline hydroxyapatite. This is clear from Figure 1, which shows a normalized sum of all background subtracted diffractograms, overlaid with the theoretical peak positions for hydroxyapatite.^19^

In addition to the continuous Debye-Scherrer rings corresponding to hydroxyapatite, discrete diffraction spots were also observed in the two-dimensional scattering patterns, indicating the presence of a minor phase with substantially larger crystallites than the nanocrystalline hydroxyapatite major phase. These diffraction spots were separated from the continuous hydroxyapatite diffraction rings, as described in the 'Materials and Methods' section, and the integrated diffractograms (Figure 1) confirm that this crystalline phase is whitlockite (a magnesium substituted calcium orthophosphate with a beta-tricalcium phosphate structure).

**Figure 2:**
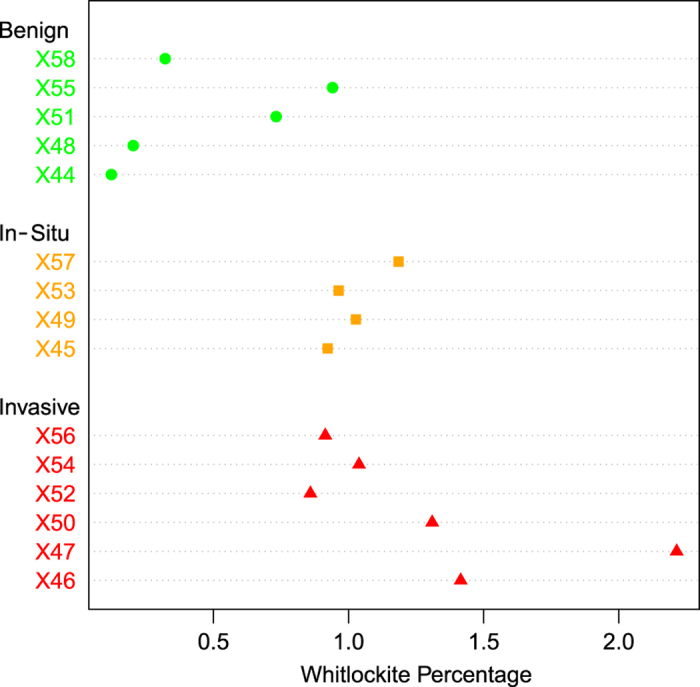
Whitlockite weight percentage average per specimen, grouped by diagnosis. Malignant specimens contain significantly more whitlockite than those with a benign diagnosis. Individual specimen details are available in Supplementary Information.

The relationship between pathology and the percentage of whitlockite within the calcifications, averaged by specimen, is shown in Figure 2. With diagnosis treated as an ordinal variable ranked ‘benign’<‘*in situ*’<‘invasive’, the percentage of whitlockite per specimen is positively correlated with diagnosis (*P*=0.008 Kendall’s tau-b), i.e., the benign specimens contain the least whitlockite and the invasive specimens contain the most.

No evidence could be found of other crystalline phases. In particular, the scatter angle region from 29° to 30° was examined for evidence of weddellite (calcium oxalate dihydrate) and brushite (calcium hydrogen phosphate dihydrate), as this region is well separated from hydroxyapatite and whitlockite peaks and contains two major (>10%) peaks each for weddellite and brushite. In a plot of the maximum intensity over all 548 diffractograms, no peaks could be observed corresponding to these two phases.

### Hydroxyapatite analysis

The analysis of positions and widths of the intensity maxima in the diffraction pattern gives some insight into both the ionic substitutions in the crystal lattice and the size of the crystallites.

**Figure 3:**
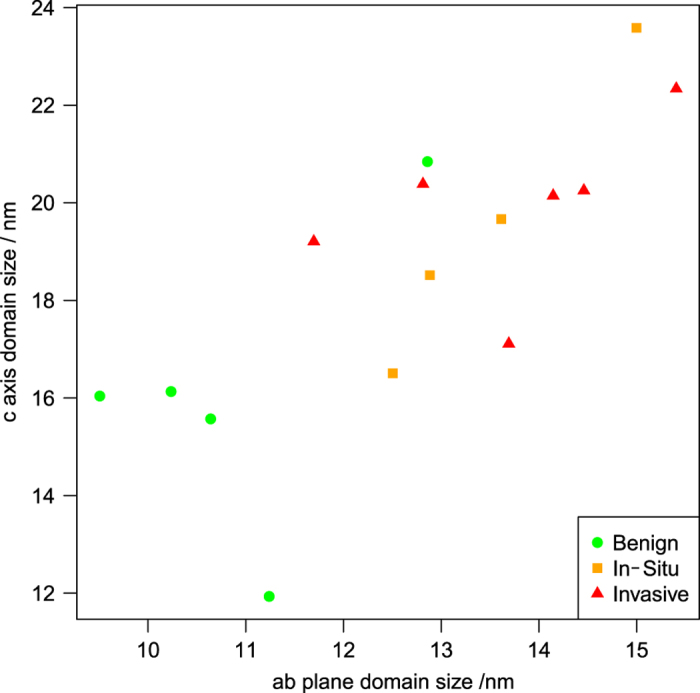
Hydroxyapatite crystalline domain dimensions, averaged by specimen and categorized by diagnosis. Malignant specimens have significantly larger crystalline domain size in both axes than benign specimens.

The hydroxyapatite crystalline domain dimensions were calculated from the fitted peak widths as described in the 'Materials and Methods' section, and are plotted in Figure 3. With the diagnosis treated as an ordinal variable ranked ‘benign’<‘*in situ*’<‘invasive’, the specimen mean crystallite domain ‘c’ axis length and ‘ab’ plane domain size both showed a positive correlation with diagnosis (*P*=0.034 and *P*=0.011, respectively, Kendall’s tau-b). With diagnosis treated as a binary variable, malignant specimens had larger ‘c’ axis length and ‘ab’ plane domain size than benign specimens (*P*=0.040 and *P*=0.005, respectively, Mann–Whitney *U*).

**Figure 4:**
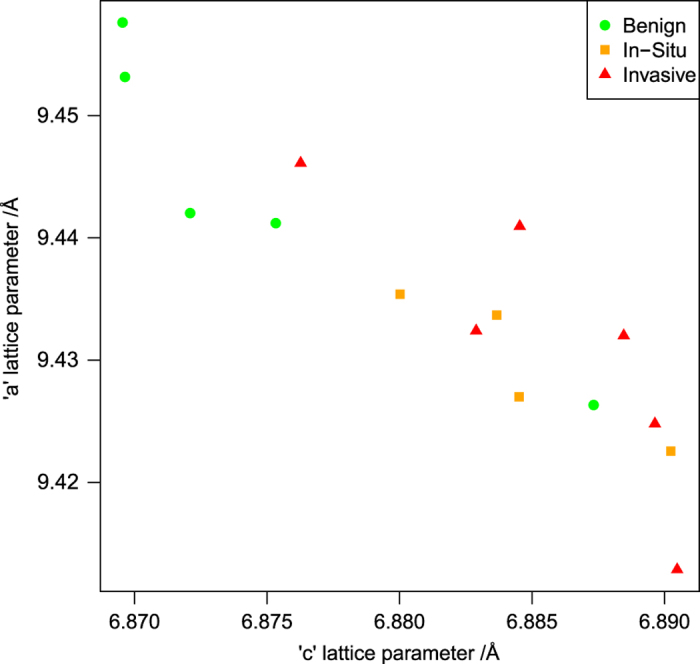
Hydroxyapatite lattice parameters, averaged by specimen and categorized by diagnosis. There is a significant negative correlation between ‘a’ and ‘c’ lattice parameters, and the ‘c’ lattice parameter is significantly smaller in benign than in malignant specimens.

The calculated ‘a’ and ‘c’ lattice parameters of the hydroxyapatite phase, averaged by specimen, are plotted in Figure 4. There is a negative correlation, with a slope of ‘a’ versus ‘c’ of −1.46 (95% confidence interval: −1.87 to −1.05). With the diagnosis treated as an ordinal variable, as in the domain size analysis, the mean specimen ‘c’ lattice parameter showed a positive correlation with diagnosis (*P*=0.025, Kendall’s tau-b). Using a binary analysis, the ‘c’ lattice parameter is larger in malignant specimens than in benign (*P*=0.019, Mann–Whitney *U*). The correlation between ‘a’ lattice parameter and diagnosis was not significant.

### Spatial variation

As the measurements were taken in uniformly spaced lines across each calcification, the position of each measurement in the sequence can be used to calculate an approximate proportion of the distance from the center to the edge. The 002 FWHM (full width half maximum) of each measurement was normalized to the mean FWHM for that calcification, and the variation from the center to the periphery calculated by linear regression. On average, the 002 FWHM is 5.4% (2.6–8.2%) greater at the edge than the center (*P*<0.001). This trend is very similar for benign, *in situ*, and invasive specimens.

## Discussion

### Major phases

The presence of calcifications consisting predominantly of nanocrystalline hydroxyapatite is unsurprising and concurs with previous X-ray diffraction characterizations. Perhaps more surprisingly, no ‘Type I’ calcium oxalate weddellite calcifications were observed in any of the 56 calcifications analyzed. In addition, no calcium oxalate was found in 236 calcifications from 110 patients in an FTIR study of samples taken from the same archive.^16^ Other studies have reported a widely varying proportion of calcium oxalate calcifications in biopsy tissue (e.g., 0.4%,^20^ 7.3%,^14^ 17.3%,^21^ 28.8% (ref. 22)). Although the specimen numbers in this pilot study were small, the absence of calcium oxalate in these results suggests a proportion towards the lower end of this range.

### Whitlockite

The presence of whitlockite in these specimens is a novel finding. It has occasionally been reported in other pathological calcifications, including prostate,^23^ aorta,^24^ cartilage,^25^ and salivary glands.^26^ A cursory mention of a single FTIR observation in a breast calcification with an absorption band at 990/cm consistent with the presence of whitlockite was reported previously.^27^ Our study is therefore the first to make a positive confirmation of whitlockite in breast calcifications.

The occurrence of whitlockite implies the presence of magnesium. Magnesium in breast calcifications has previously been reported in one study,^22^ with the proportion of specimens containing magnesium increasing from benign to *in situ* to invasive. This is consistent with our X-ray diffraction results showing the least whitlockite in benign specimens, and the most in invasive carcinoma. The presence of magnesium whitlockite is potentially of significance as several studies have shown that magnesium levels both influence and are influenced by the process of carcinogenesis.^28^ In particular, significantly lowered serum magnesium levels have been found in breast cancer patients,^28^ and it has been suggested that a high Ca:Mg ratio is risk factor in the development of postmenopausal breast cancer.^29^

At first sight it is therefore surprising to find a higher concentration of whitlockite in calcifications within malignant specimens. However, a peculiarity of tumor cells is their avidity for Mg, which accumulates within the cells even when extracellular Mg concentrations are low.^29^ Whether calcifications form from the products of cell necrosis or through matrix vesicles,^30^ raised intracellular magnesium is likely to elevate the level of magnesium within calcifications associated with breast cancer.

The observation of more magnesium whitlockite in malignant than benign tissue specimens, together with other evidence for higher levels of magnesium in malignant calcifications,^22^ may provide a clue as to why carbonate levels are lower in calcifications associated with cancer. It has recently been shown that magnesium ions can strongly inhibit the incorporation of carbonate in hydroxyapatite.^31^ Increased concentration of magnesium within cancer cells could lead to higher magnesium levels within calcifications, leading in turn to lower carbonate levels in calcifications associated with breast cancer.

### Precursor phases

Both brushite and OCP (octacalcium phosphate) have been identified as precursors to the formation of hydroxyapatite in mineralized tissue, particularly in an acidic environment,^32^ which is typical of the microenvironment of solid tumors.^33^ No brushite was observed in these specimens. OCP could not be distinguished from hydroxyapatite by X-ray diffraction in this study, as the patterns are nearly identical over the diffraction angular range captured. The most prominent differentiating sign is the strong low angle OCP (1 0 0) peak with a d-spacing of 19.7 Å, which does not occur in hydroxyapatite. However, this diffraction peak occurs considerably below the lowest angle collected in this study, and would require a different experimental setup to detect. The presence of OCP cannot therefore be confirmed or ruled out in these specimens.

### Comparison with maturing bone

It has been suggested that the mechanisms involved in the formation of breast calcifications may be similar to those involved in the deposition of hydroxyapatite in bone; in particular, bone matrix proteins involved in osteoblast mineralization are also expressed in mammary cells.^30^ It is therefore interesting to compare the observed characteristics of breast calcifications with those of bone in varying stages of maturity. Calcifications in specimens with a benign diagnosis have smaller crystalline domains, a smaller ‘c’ lattice parameter, and a larger ‘a’ lattice parameter than specimens with *in situ* or invasive carcinoma. Hydroxyapatite crystallite domain size along the ‘c’ axis in human fetal bone has been shown to increase with increasing gestational age, accompanied by an increase in c/a lattice parameter ratio.^34^ This increase in crystallite domain size dimensions continues up to the age of about 20, also accompanied by an increase in ‘c’ lattice parameter and decrease in ‘a’ lattice parameter.^35^ This combination of changes in lattice parameters and crystalline domain size with age in both fetal and child bone parallels our observations in breast calcifications from benign to *in situ* to invasive. The mean ‘c’ axis domain size in benign breast calcifications of 10.9 nm is approximately equivalent to 17 week gestation fetal bone, whereas in invasive specimens, the mean ‘c’ axis domain size of 13.7 nm is approximately equivalent to bone at 23 weeks gestation. In addition, the mean c/a ratio of 0.728 in benign specimens is similar to that of fetal bone at gestational age of 16 weeks, whereas in invasive specimens, the ratio of 0.730 is similar to that of bone at 25 weeks gestation. Of course, these similarities do not imply a temporal progression in the breast calcifications, but may reflect comparable differences in chemistry.

## Materials and Methods

### Archive blocks

Formalin-fixed paraffin-embedded core biopsy breast specimens were selected from the Gloucestershire Hospitals NHS Foundation Trust diagnostic archive (under approval from the Gloucestershire Local Research Ethics Committee). The blocks were randomly selected from the 2012 archive, subject to the presence of calcifications in the histopathology report, and an unambiguous classification of ‘B2—Benign’, ‘B5a—Ductal Carcinoma *In Situ*’, or ‘B5b—Invasive Carcinoma’.^36^ For the purposes of analysis, the specimens were also grouped as ‘Benign’ (B2) versus ‘Malignant’ (B5a or B5b). A summary of the histopathology report for each of the specimens analyzed is given in Supplementary Table S1. These specimens were screened by mounting the blocks in a Nikon Metrology XT H225 CT system and imaging at 20 kV both perpendicular and parallel to the face of the block. Following screening, the blocks were selected with significant levels of calcification at or near the cut surface. The selected blocks were then CT-scanned, and the reconstructed volumes processed as previously described,^37^ to identify the position of calcifications relative to the tissue outlines at the surface of the blocks.

### Mounting

**Figure 5:**
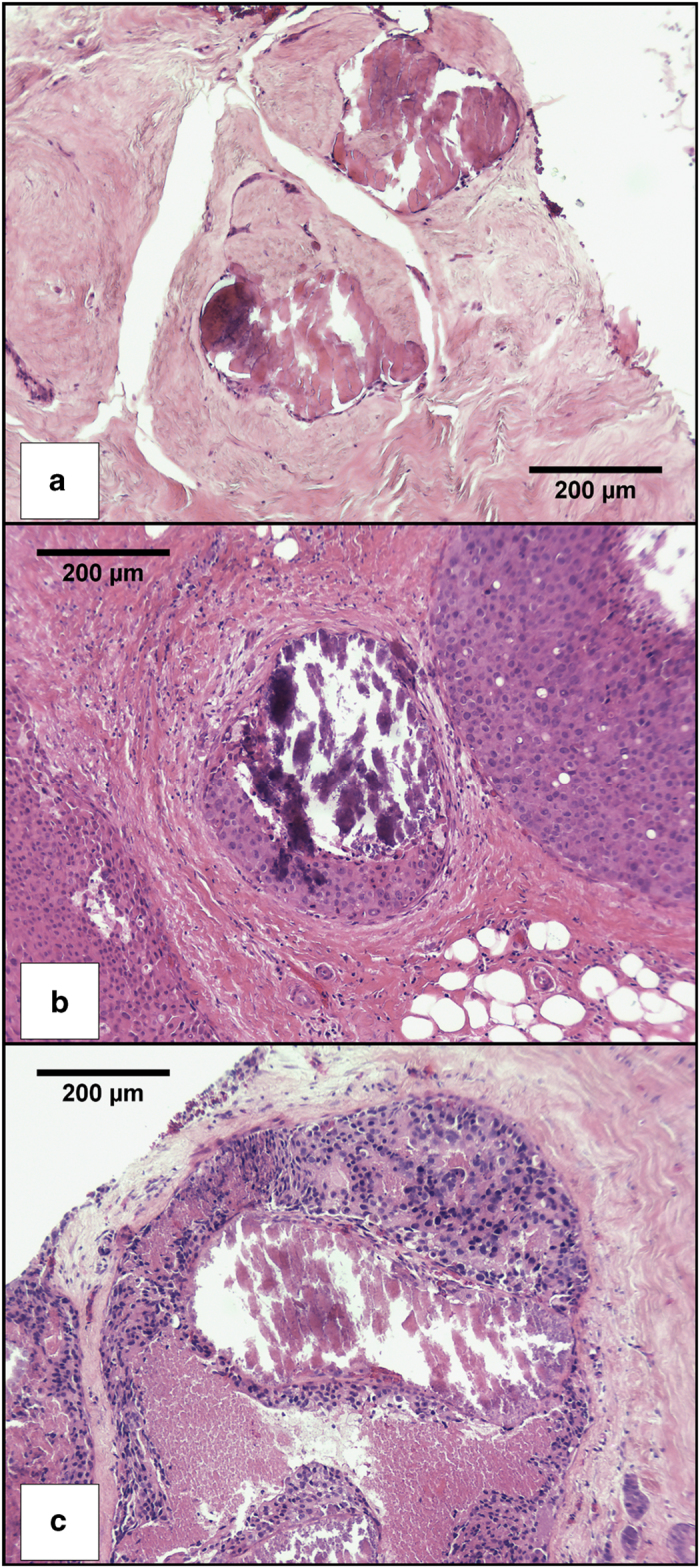
H&E stained images of typical calcifications: (**a**) Specimen X44 (B2), (**b**) Specimen X53 (B5a), (**c**) Specimen X46 (B5b). Histopathology summaries can be found in Supplementary Table S1.

Two sequential microtome slices of 5 μm were cut from each block. The first slice underwent standard H&E (hematoxylin and eosin) processing, and was used for reference. Figure 5 shows typical H&E images of calcifications within specimens classified as B2, B5a, and B5b. The second slice was mounted on a 12.5 μm thick polyolefin heat shrink film that was stretched taut over a 38 mm diameter aluminum alloy ring, and held in place with an ‘O’ ring in a groove. This film was chosen as it only exhibits weak diffraction lines, which are away from peaks of interest in candidate calcification phases. In addition, these lines are the same position as those from the paraffin-embedding material.

NIST (National Institute of Standards & Technology) Standard Reference Material 640c Silicon Powder was used for the calibration of sample–detector distance. A slurry was made with the standard powder in a dilute solution of PVA adhesive in distilled water, and three spots painted on the mounting film around and in the plane of the tissue section.

### Beamline setup

The X-ray diffraction experiments were conducted on beamline I18 at Diamond Light Source, Didcot, UK. Although in principle X-ray diffraction measurements could have been conducted using a laboratory source, synchrotron radiation offers substantial advantages for this type of research. First, the high brilliance possible with synchrotron radiation enables measurements to be made on particles such as breast calcifications, substantially quicker than even a high brightness laboratory source. Second, instrumental broadening is typically insignificant relative to sample broadening in this type of specimen when using synchrotron diffraction, which minimizes a potential confounder when performing peak-broadening analysis. Third, we wished to investigate the variation in characteristics within individual calcifications, which requires a spatial resolution that is only realistically achievable with a microfocus synchrotron source.

The aluminum sample rings were clamped perpendicular to the beam on a motorized stage. Measurements were made in transmission using a Photonic Science X-ray SCMOS camera. A video microscope was mounted at 45° to the beam and used to position the desired point on the tissue section in the beam. A beam spot size of 10×10 μm was used, with an energy of 10.0 keV. The specimen-to-detector distance was kept constant by ensuring the center of the microscope field of view was in focus at maximum magnification. This was confirmed by measurements on each of the three silicon standard dots on every specimen. Calcifications were located using the video microscope, by reference to the visible tissue outlines and the maps created from the CT measurements. The measurements were made on typically 11 equally spaced positions in a vertical line from the bottom to the top of each calcification. Exposure was for 30 s per measurement. Data acquisition was performed using the Diamond Generic Data Acquisition software, with images and ancillary data saved in NeXus data file format. A total of 548 diffractograms were collected from 56 discrete calcifications in 15 specimens, consisting of 5 benign, 4 *in situ*, and 6 invasive. In addition, diffractograms were collected for the silicon standards surrounding the specimens. These sample sizes are sufficient to achieve significant group differences using nonparametric testing.

### Analysis

Azimuthal integration of the two-dimensional diffraction images to one-dimensional patterns was conducted using DAWN.^38^ The silicon standard images were used for calibration of beam center, detector tilt, and sample–detector distance.

Diffractograms exhibited a large background-to-signal ratio, and it was found beneficial to subtract the background as a separate step before pattern fitting. The background was subtracted in R^39^ using the Peak Filling method,^40^ with the background centered in the noise band. Following background subtraction, pattern fitting was conducted using TOPAS 4.2 software (BRUKER AXS).

The whitlockite diffraction spots were separated from the continuous hydroxyapatite rings by cake-remapping in DAWN, followed by azimuthal background subtraction and azimuthal integration at each 2*θ* value in R. This is similar to a method recently proposed for the detection of abnormal grain growth in metals.^41^

The average percentage of whitlockite was calculated for each specimen from the total area of the two strongest whitlockite peaks, (0 2 10) and (2 2 0) at 2*θ*≈25.05° and 27.8°, respectively, and from the total area of the overlapping (2 1 1), (1 1 2), (3 0 0), and (2 0 2) hydroxyapatite peaks that lie in between. Those areas were converted to weight percent using Reference Intensity Ratios of Hydroxyapatite and whitlockite from the ICDD PDF-2 database.^42^ However, it should be noted that the small number of whitlockite crystallites in a favorable orientation in each diffractogram will lead to inaccuracies in the relative area of each peak in the pattern.

The hydroxyapatite crystalline domain size was calculated from the fitted FWHM of the (002), (102), (210), (211), (112), (300), (202), (310), (222), (312), (213) peaks. A Bayesian approach was taken to decomposition of the heavily overlapped (211), (112), (300), (202) peaks. This involved a least-squares fit using fixed peak positions. These positions were calculated for each diffractogram from the ‘a’ and ‘c’ lattice parameters from the whole pattern fit. As is typical of synchrotron measurements on this type of material, instrumental broadening was negligible relative to the broad diffraction peaks, and was neglected. All FWHM measurements were converted to Integral Breadth using the Lorenzian mixing factor of the fitted pseudo-Voigt peaks, and aggregated at specimen level as a weighted mean, using total peak area as a weight. Hydroxyapatite crystallite shape was modeled as a cylinder with its axis in the ‘c’ direction. The apparent coherent domain thickness in the direction of each diffraction vector was plotted in a polar plot against the angle of the reflecting plane to the crystallographic ‘c’ axis. A least-squares fit line from the Langford cylindrical model^43^ was fitted to the data points, and the best-fit cylinder length and diameter from the model used as the dimensions of the coherently diffracting crystallite domains.

Inhomogeneous lattice strain from ionic substitution undoubtedly also contributes to peak broadening, and in theory it is possible to separate this from physical size effects by Williamson–Hall or Warren–Averbach methods. However, this is complicated by the fact that peak broadening in this case is markedly anisotropic, and the separation of anisotropic strain from anisotropic size effects requires data quality that is difficult to achieve with biological apatite. As a result of this, and other unaccounted sources of peak broadening, the calculated size of the coherently diffracting crystallite domains is bound to be smaller than the physical size of the crystallites. Indeed, it has been suggested that bone mineral is better described as paracrystalline, with a continuum of overlapping domains rather than crystallites with definite crystal boundaries.^44^ Using coherently diffracting domain size, uncorrected for inhomogeneous strain, ensures comparability with the majority of studies on bone.
