# Supplementary material for: Relationships between pathology and crystal structure in breast calcifications: an in situ X-ray diffraction study in histological sections
Source: NPJ Breast Cancer. 2016 Sep 14;2:16029–. doi: 10.1038/npjbcancer.2016.29 (PMC5515336; doi:10.1038/npjbcancer.2016.29)
Supplement: Supplementary Information [file npjbcancer201629-s1.doc]

**Relationships between pathology and crystal structure in breast calcifications: an in-situ X-ray diffraction study in histological sections**

Supplementary Information – Table S1

| **Specimen** | **Histological Opinion** | **# Cores** | **Micro. Report: Summary extract** | **Whitlockite %** | **Domain size / nm** | | **Lattice Parameters /‎Å** | |
| --- | --- | --- | --- | --- | --- | --- | --- | --- |
|  |  |  |  |  | **‘ab ’** | **‘c’** | **‘a’** | **‘c’** |
| X44 | B2 | 5 | Fibroadipose breast tissue including a fibroadenoma. Dystrophic calcification is noted within the fibroadenoma. There is no cytological atypia. | 0.12% | 10.24 | 16.13 | 9.4576 | 6.8695 |
| X45 | B5a | 1 | Intermediate and high nuclear grade DCIS associated with malignant microcalcification. DCIS shows solid cribriform and papillary growth patterns. There is no evidence of invasive malignancy. | 0.92% | 12.88 | 18.52 | 9.4337 | 6.8837 |
| X46 | B5b | 4 | High nuclear grade DCIS associated with malignant microcalcification and invasive ductal carcinoma which appears to be moderately differentiated. Immunohistochemistry for oestrogen receptors demonstrates that more than 67% of neoplastic cells of this tumour show strong nuclear positivity, giving a Quick score of 8/8. | 1.41% | 15.41 | 22.34 | 9.4248 | 6.8896 |
| X47 | B5b | 2 | These fragments of breast tissue bear infiltrating carcinoma, on this evidence moderately differentiated ductal carcinoma comprising cohesive nests and cords of cells infiltrating hyalinised fibroelastotic stroma. The tumour does show some lobular features. In addition there is high nuclear grade DCIS of solid and cribriform types with comedo necrosis and associated malignant calcification. Immunostaining for oestrogen receptors shows the tumour to be strongly ER positive (quick score 8/8). The tumour is E-Cadherin positive supporting the diagnosis of ductal carcinoma. | 2.21% | 13.69 | 17.11 | 9.4320 | 6.8885 |
| X48 | B2 | 5 | These biopsies are of breast tissue showing dense stromal sclerosis associated with coarse calcification which appears dystrophic. The epithelial elements show columnar cell change and mild usual type hyperplasia. There is no evidence of atypia or of in-situ or invasive malignancy in these biopsies. | 0.20% | 11.24 | 11.93 | 9.4420 | 6.8721 |
| X49 | B5a | 3 | These breast core biopsies show intermediate nuclear grade ductal carcinoma in-situ of cribriform type with apocrine features. There is associated malignant microcalcification. There is no evidence of invasion in these biopsies. | 1.03% | 12.50 | 16.50 | 9.4354 | 6.8800 |
| X50 | B5b | 2 | Cores of sclerotic and elastotic stroma which are infiltrated by a ductal carcinoma. The tumour appears well differentiated with well formed ducts lined by bland epithelial cells. There is much micro-calcification associated with the lesion and a small in-situ component is also present. Immunohistochemistry shows strong nuclear positivity in almost all tumour cells for oestrogen receptor giving a Quick score of 8/8. | 1.31% | 14.46 | 20.25 | 9.4129 | 6.8905 |
| X51 | B2 | 5 | The specimen comprises rather fragmented cores of benign breast tissue exhibiting a normal tubulo-lobular architecture with quite marked hyaline fibrosis of the stroma. There is some evidence of fibrosis of the intra-lobular stroma suggesting some fibrocystic change but some of the fragments show fibroadenomatoid features comprising expanded hyaline hypocellular stroma within which, there are some curvilinear compressed ducts. This fibroadenomatoid tissue is associated with large foci of micro-calcification. Although appearances may represent fibroadenomatoid change, part of a fibroadenoma is also a possibility. There is no evidence of malignancy. | 0.73% | 9.51 | 16.04 | 9.4532 | 6.8696 |
| X52 | B5b | 2 | These core biopsies are infiltrated by a well differentiated ductal carcinoma (T1 P2 M2). There is associated microcalcification and focal DCIS. Immunohistochemistry for oestrogen receptors demonstrates that more than 67% of neoplastic cells of this tumour show strong nuclear positivity, giving a Quick score of 8/8. | 0.86% | 11.70 | 19.21 | 9.4461 | 6.8763 |
| X53 | B5a | 7 | Histological assessment reveals that both specimens consist of cores of breast tissue within which there is high nuclear grade ductal carcinoma in-situ with a solid growth pattern associated with comedo necrosis and malignant micro-calcification. In areas, this has the features more in keeping with pleomorphic lobular carcinoma in-situ (LCIS) but, from a management point of view, should be regarded as DCIS. There is a some scarring and inflammation associated with some of the ducts but there is no definite evidence of invasive malignancy, although, in specimen 2, is this more concerning and immunohistochemistry will be carried out and a supplementary report will be issued. SUPPLEMENTARY: Immunohistochemistry has been carried out. There is no evidence of invasive malignancy. The in-situ component is negative for E-cadherin. This is considered to pleomorphic lobular carcinoma in-situ (LCIS). From a management point of view, it should be treated as for DCIS. The categorisation of B5a remains. | 0.96% | 13.61 | 19.67 | 9.4270 | 6.8845 |
| X54 | B5b | 1 | A core of desmoplastic fibro-elastotic stroma bearing infiltrating adenocarcinoma, on this evidence moderate to poorly differentiated ductal carcinoma. Some probable high nuclear grade DCIS showing comedo necrosis is seen and there is malignant calcification. Immunostaining for oestrogen receptors has been performed (1) revealing the tumour to be strongly ER positive (quick score 8/8). | 1.04% | 14.15 | 20.15 | 9.4409 | 6.8845 |
| X55 | B2 | 5 | Stereo-cores right breast, representative calcs seen: This specimen comprises benign breast tissue exhibiting a normal tubulo-lobular architecture. There are well established fibrocystic changes with areas of fibrosis of the inter and intra-lobular stroma with dilatation and separation of acini. Apocrine and lactational type change is seen and in some areas, there is columnar cell change associated with benign micro-calcification. There is some cross cutting of the ducts. The specimen has been examined on multiple levels and neither in-situ nor infiltrating neoplasia is seen. | 0.94% | 12.86 | 20.84 | 9.4263 | 6.8873 |
| X56 | B5b | 1 | This core biopsy shows a grade 1 infiltrating ductal carcinoma, not otherwise specified (tubules 1, pleomorphism 2, mitosis 1). Malignant microcalcifications are seen. There is no convincing DCIS. ER Quick score is 7/8 (5 + 2). | 0.91% | 12.81 | 20.38 | 9.4324 | 6.8829 |
| X57 | B5a | 3+2 | 1 and 2: Biopsies from both sites consist of breast tissue showing similar features with intermediate nuclear grade ductal carcinoma in-situ showing apocrine features. There is associated comedo necrosis with malignant microcalcification. There is no evidence of invasive malignancy in either specimen. | 1.19% | 15.00 | 23.59 | 9.4226 | 6.8902 |
| X58 | B2 | 9 | These core biopsies show features of fibrocystic change with many areas of sclerosing adenosis and focal benign microcalcification. There is no evidence of epithelial hyperplasia, in situ or invasive malignancy. | 0.32% | 10.64 | 15.57 | 9.4412 | 6.8753 |
